# Belief in Food Addiction and Obesity-Related Policy Support

**DOI:** 10.1371/journal.pone.0147557

**Published:** 2016-01-25

**Authors:** Erica M. Schulte, Hannah M. Tuttle, Ashley N. Gearhardt

**Affiliations:** 1 Department of Psychology, University of Michigan, Ann Arbor, Michigan, United States of America; 2 Center for Developmental Science, University of North Carolina, Chapel Hill, North Carolina, United States of America; University of Bath, UNITED KINGDOM

## Abstract

**Objectives:**

This study examines whether belief in the food addiction construct is associated with support for obesity-related policies (e.g., restrictions on foods served in schools and workplace cafeterias, subsidies on fruits and vegetables), while simultaneously examining other factors associated with policy support (e.g., political party affiliation).

**Design:**

Cross-sectional.

**Setting:**

Online Community.

**Participants:**

200 individuals were recruited through Amazon Mechanical Turk.

**Measurements:**

Participants (n = 193) responded to three questions about belief in food addiction and a measure evaluating support for 13 obesity-related policy initiatives. Individuals also completed the modified Yale Food Addiction Scale (mYFAS), self-reported height and weight, and provided demographic information (age, gender, race, political party affiliation).

**Results:**

Belief in food addiction was significantly associated with greater support for obesity-related initiatives, even when accounting for the significant associations of age, gender, and political party. Belief in food addiction and political party both had moderate effect sizes for predicting support for obesity-related policy. There was an interaction between age and belief in food addiction, with significant associations with policy support for both younger and older individuals, though the effect was larger for younger participants.

**Conclusion:**

The current study provides evidence that belief in food addiction is associated with increased obesity-related policy support, comparable to the influence of one’s political party. Growing evidence for the role of an addictive process in obesity may have important implications for public support of obesity-related policy initiatives.

## Introduction

Food addiction is a topic of public interest and scientific debate [1, 2]. Evidence for the hypothesis that some individuals may experience an addictive-like response to certain foods is growing. Animal model studies observe that consumption of high-sugar, high-fat foods (e.g., cheesecake, Oreos) is associated with neurobiological changes in the reward system, such as the downregulation of dopamine receptors, and indicators of addiction, such as increased motivation, withdrawal, and tolerance [3–5]. In humans, obesity and addiction are associated with similar patterns of dysfunction in reward-related and executive control neural systems [6, 7]. Eating-related problems (e.g., obesity, binge eating) and addictive disorders share behavioral characteristics, such as a loss of control over consumption and continued use despite negative consequences [8–10]. Further, highly processed foods with added fat and refined carbohydrates (e.g., chocolate, pizza, chips) are particularly associated with behavioral indicators of addictive-like eating [11]. Yet, the specific addictive agent in food has not been investigated, which remains one of the major points of criticism for the food addiction hypothesis [2] Though more research is warranted to identify the addictive ingredient(s) in highly processed foods and the individual characteristics that may make one vulnerable to developing food addiction, behavioral and biological evidence exists for the concept of addictive-like eating.

Historically, the identification of a substance as addictive shifts public perceptions in a manner that increases support for public policies that aim to reduce the negative impact of the substance (e.g., restrictions on marketing, taxation) [12]. For example, the identification of nicotine as addictive, rather than habit forming, was one of the defining moments that shifted public attitudes about cigarettes and led to the development of new tobacco-focused policies [13, 14]. However, it is unknown whether the application of a food addiction framework or the identification of certain foods as addictive will impact support for policy initiatives targeting eating-related problems like obesity [15] in a similar manner as nicotine, especially given key differences with food (e.g., no direct harm from second-hand smoke). Previous studies suggest that food addiction is publically perceived as a contributor to obesity. In a sample of Australian and American adults, 86% of participants reported that certain foods, especially those high in sugar, are addictive, and 80% of individuals believed these foods could be as addictive as substances like alcohol, cigarettes and cocaine [16]. Further, 72% of participants endorsed the idea that food addiction is a cause of obesity [16]. In a qualitative study of low-income women, food addiction was described in a “matter of fact” way and understood to be a compulsive need for certain foods [17]. While the public appears to accept that certain foods may be addictive and food addiction is a causal explanation for obesity, there has been little examination of how an addiction framework may impact food policy support.

It is possible that applying an addiction perspective to the obesity epidemic may be ineffective or even harmful. In a recent paper, Rasmussen [18] reviewed the historical context of the 1950s when addiction-based explanations for obesity were common. This framework was related to more negative views of individuals with obesity and an emphasis on psychoanalytical group therapy, rather than population-level policy, to reduce the public health costs of obesity [18]. Yet, the understanding of addiction has changed markedly in the last sixty years. The modern conceptualization of addiction is biobehavioral and emphasizes that the combination of individual risk factors (e.g., genetics, age of first use) and exposure to an addictive substance or behavior (e.g., cocaine, gambling) can trigger neuroplastic changes in brain systems implicated in compulsive behavior (e.g., mesolimbic dopaminergic system) [19–21]. Though it has been suggested that this model may not change stigma [22], or even increase it [23], by labeling individuals as “diseased” or lacking control and overemphasizing the medicalization of treatment [24], it is also plausible that a biobehavioral perspective will decrease stigma by reducing blame on the individual and increasing funding for intervention research [25]. Notably, an individual’s beliefs about personal responsibility more generally (e.g., controllability of poverty, obesity, etc.) may contribute to both one’s political party orientation and level of stigma towards individuals with obesity or an addictive disorder [26, 27].

One previous study found that an individual’s endorsement of food addiction symptoms was not associated with their reported levels of weight-related stigma [28]. However, Latner and colleagues [29] observed that exposure to explanations of obesity emphasizing an addiction perspective, relative to explanations focusing on genetic and homeostatic factors, was related to less weight stigma towards obese individuals. There has been limited examination of how an addiction framework may influence food consumption. One recent study did not observe a relationship between exposure to a message that food addiction is real (relative to a message that food addiction is myth) and mean food intake [30]. Though more research is warranted on how an addiction perspective may impact eating behavior, an emphasis on addiction as a contributor to eating-related problems may reduce negative views of individuals with obesity. Further, as was the case with nicotine, it is plausible that public perceptions of highly processed foods as addictive may be related to greater support for obesity-focused policy approaches. One previous study found that support for food addiction was not associated with perceived effectiveness of various policies (e.g., education and support programs, restrictions on food advertising) for the treatment of food addiction [16]. However, no prior studies have examined how belief in food addiction may be related to support for the implementation of obesity-related policies.

The current study will also explore whether other factors are predictive of obesity-related policy support. While the public as a whole appears to favor educational approaches over government-based interventions for obesity [31], individual characteristics are predictive of differential support. Women and Democrats indicate increased levels of support for policies that limit access to highly processed foods, such as taxes on sugar-sweetened beverages and regulating television marketing to children [31–33]. Similarly, older individuals are more supportive of restriction-based interventions (e.g., removing vending machines from schools) than younger (18-year-old) individuals [32]. Additionally, minority racial groups, parents, and individuals with higher BMIs endorse greater support for policy initiatives targeting children, such as banning the sales of fast-food items in schools [32]. Thus, in order to understand whether belief in food addiction influences public opinion of food policy initiatives, it is essential to also examine participant-specific predictors of obesity-related policy support.

The current study examines the association between public belief in food addiction and support for obesity-related policy. It is hypothesized that individuals who endorse their belief in the food addiction construct will exhibit greater support for food policy initiatives. Further, we will explore whether individual characteristics (age, gender, race, food addiction symptomology, BMI, and political party affiliation) are 1) associated with policy support, 2) associated with belief in food addiction, and 3) moderate the relationship between food addiction belief and policy support.

## Methods

### Ethics Statement

The University of Michigan Health and Behavioral Sciences Institutional Review Board approved the current study (HUM00089673) and written informed consent was obtained from all participants.

### Participants

Participants (*n* = 200) were recruited online through Amazon’s Mechanical Turk (MTurk) worker pool and restricted participation to MTurk users in the United States. While MTurk’s sample is not nationally representative, diversity in participant demographics is comparable to traditional convenience samples [34]. All participants were over the age of 18 and provided written consent to participate in a study about food and policy. Individuals were compensated $.30 for their time, which is a rate consistent with other MTurk studies [34]. Participants (*n* = 7) were excluded from data analysis for incorrectly answering “catch” questions. These questions have well-known answers (e.g., “What does 2+2 equal?”) and are included in the study to catch individuals responding without carefully reading the questions.

Participants ranged in age from 18–73 years *(M* = 36.95, *SD* = 12.41) and 58.5% (*n* = 113) were female. Individuals’ self-reported racial identification was as follows: 81.9% White, 6.7% Asian/Pacific Islander, 4.7% African American, 3.1% Hispanic, and 3.6% Other. BMI was calculated from self-reported height and weight and ranged from underweight (16.98) to obese (50.21), with an average BMI in the overweight category (M = 26.70 and *SD* = 6.16). The BMI distribution by weight class for the sample was as follows: 44.3% normal weight, 27.1% overweight, 26.0% obese and 2.6% underweight. Participants also reported political party identification, and 38.9% identified as Democrats, 29% Independents (unaffiliated with a particular party), 17.1% Republicans, 11.4% No Affiliation/Unsure, and 3.6% Libertarians. Libertarians and Republicans did not significantly differ in both their belief in food addiction or their support for obesity-related policies (*p*s > .57), and theoretically, both parties tend to be more conservative in nature relative to Democrats [35]. Thus, these political orientations were combined for analyses given the sample size of Libertarians (*n* = 7).

### Assessments and Measures

Participants completed surveys to examine their beliefs about food addiction, support for obesity policy initiatives, addictive-like eating behavior, and individual characteristics.

#### Belief in food addiction

Adapted from procedures by Latner and colleagues [29], participants responded to the following three statements about the addictive nature of food: 1) “Food has addictive properties, like a drug,” 2) “Body weight can result from being addicted to food,” and 3) “Obesity can result from being addicted to food.” A six-point Likert scale was used to assess participants’ opinions (1 = strongly disagree to 6 = strongly agree). Latner and colleagues [29] reported good internal consistency for these three items (α = .86) and utilized the total score as an “addiction rating.” Similarly, the current study used the composite score of the three items to reflect belief in food addiction, which had good internal consistency (α = .89). In the current sample, participants’ belief in food addiction ranged from 0 to 15 (*M* = 10.72, *SD* = 3.72). Additionally, 49.7% of individuals reported that they moderately to strongly agreed with all three statements assessing belief in food addiction.

#### Support for Obesity-Focused Policy Initiatives (SOPI)

A 13-item questionnaire was developed to assess participants’ level of support for certain food-related policies. Sample items include: “I support insurance companies providing coverage for psychological treatment for eating-related affiliations” and “I believe that restaurants should be required to display nutrition content of food and beverage products they serve” (see Table 1 for the full list). Items were developed through a literature review of previous studies examining obesity-related policies [16, 31–33]. Participants were asked to rate their support for each of the statements on a five-point Likert scale (0 = strongly disagree to 5 = strongly agree). An exploratory factor analysis revealed four potential factors with an eigenvalue greater than one (5.13, 1.30, 1.07, 1.03), however plotting the factors suggested a single factor structure that accounted for 39.48% of variance in SOPI responses. The scale also demonstrated good internal consistency (α = .87) and split-half reliability (*ρ* = .85). Thus, the current study utilized a summary score of all 13 items to indicate obesity-related policy support. Participants’ total obesity-related policy support ranged from 3 to 65 (*M* = 43.10, *SD* = 12.31) (see Table 2 for mean levels of support and standard deviation for individual policies).

**Table 1:** Support for Obesity-Related Policy Initiatives (SOPI) Questions^1^.

1. I support restrictions on the size of fountain drink cups served in fast-food restaurants and convenience-style stores.
2. I believe that restaurants should be required to display nutrition content of food and beverage products they serve.
3. I believe there should be restrictions on the type of foods served in K-12 schools.
4. I believe there should be restrictions on the type of foods served in workplace cafeterias.
5. I support insurance companies providing coverage for psychological treatment for eating-related afflictions.
6. I support the inclusion of calorie labels on restaurant menus.
7. I believe there should be restrictions on the type of foods that can be advertised to children.
8. I believe there should be policies to prevent people from being discriminated against because of their weight.
9. I support fruits and vegetables being subsidized by the government so they are cheaper.
10. I would support a government-sponsored public service campaign that advertises the addictive qualities of sugar and other processed foods.
11. I support the repeal of government subsidies for sugar, fat, and other ingredients implicated in obesity.
12. I support subsidies to make gym memberships easier to afford.
13. I believe there should be policies to require a minimum amount of physical activity in schools.

^1^Participants responded to each statement on a 6-point Likert scale ranging from 0 (“Strongly Disagree”) to 5 (“Strongly Agree”)

**Table 2:** Support for Individual Obesity-Related Policies on the SOPI^1^.

SOPI Policy	Mean	SD
Inclusion of calorie labels on menus	4.10	1.18
Restaurants displaying nutrition information	3.92	1.37
Policies requiring physical activity minimums in schools	3.88	1.38
Insurance coverage for psychological treatment for eating-related afflictions	3.84	1.21
Restrictions on foods in K-12 schools	3.72	1.45
Government subsidies for fruits and vegetables	3.65	1.59
Policies to prevent weight-related discrimination	3.60	1.45
Subsidies for gym memberships	3.32	1.67
Repeal of government subsidies for sugar, fat, and other ingredients implicated in obesity	3.09	1.50
Restrictions on food advertisements to children	3.08	1.65
Public service campaign advertising the addictive qualities of sugar and other processed foods	2.99	1.68
Restrictions on foods in workplace cafeterias	1.97	1.71
Restrictions on fountain drink sizes	1.93	1.84

^1^Participants responded to each statement on a 6-point Likert scale ranging from 0 (“Strongly Disagree”) to 5 (“Strongly Agree”)

#### Modified Yale Food Addiction Scale (mYFAS)

The Modified Yale Food Addiction Scale (mYFAS) is an abbreviated version of the Yale Food Addiction Scale (YFAS), which utilizes DSM-IV criteria for substance dependence to operationalize addictive-like eating behaviors (e.g., loss of control, continued use despite negative consequences) [36]. The mYFAS is a self-report measure that contains nine questions: one question for each of the seven diagnostic criteria for dependence and two items to assess clinically significant impairment and distress. Flint and colleagues [37] report the mYFAS has similar psychometric properties as the full YFAS, including identical internal consistency. In the current study, the mYFAS had adequate internal consistency for such a brief measure (α = .69). Akin to the YFAS, scoring for the mYFAS includes a “symptom count” total ranging from 0 to 7, reflecting the number of dependence criteria reported. In the current sample, mYFAS symptoms ranged from 0 to 6 (*M* = 1.09, *SD* = 1.34).

#### Data analytic plan

Data analysis was performed in IBM Statistical Product and Service Solutions (SPSS) Statistics, version 21.0 (SPSS, INC., Chicago, IL). The associations of participant-specific variables with obesity-related policy support and belief in food addiction were examined utilizing one-way analysis of variance (ANOVA) techniques for categorical variables (Levene’s tests were conducted to examine homogeneity of variance, all *p*s > 0.10) and correlation analyses were used for continuous variables. Univariate general linear model (GLM) techniques were used to examine whether belief in food addiction was significantly associated with obesity-related policy support while including other factors potentially related to policy support in the model (age, gender, race, political party identification, BMI, mYFAS symptom count). In addition to main effects, interactions were also examined between belief in food addiction and other significant predictors of policy support (political party, age, and gender). All significant interactions were investigated further using the simple slopes procedures identified by Aiken and colleagues [38]. Effect sizes (partial eta squared) were computed for all GLM tests and multiple-test corrected post-hoc procedures were conducted on significant omnibus tests.

## Results

### Support for Obesity-Related Policies: Associated Individual Characteristics

Age was negatively correlated with policy support (r = -.18 *p* < .05) (Table 3) with younger individuals exhibiting greater support. One-way ANOVA tests revealed that policy support also differed by political party identification (F(3,189) = 12.72, *p* < .001, η_p_^2^ = .17). Tukey HSD tests were conducted on all possible contrasts to examine which groups had significantly different means. These post hoc analyses revealed that Democrats (*M* = 48.11, *SD* = 10.62) endorsed significantly greater obesity-related policy support relative to Republicans/Libertarians (*M* = 34.45, *SD* = 13.45) (*p* < .001), and Independents (*M* = 42.91, *SD* = 10.00) (*p* < .05). Race, gender, BMI and mYFAS symptom count were not significantly associated with policy support (all *p*s > .06).

**Table 3:** Correlations Between Policy Support and Continuous Individual Characteristics.

	Policy Support	Age	BMI	mYFAS Symptom Count
Policy Support	1	-.18*	.01	.06
Age		1	.11	.11
BMI			1	.33**
mYFAS Symptom Count				1

*Correlation is significant at the *p* < .05 (two-tailed) level

**Correlation is significant at the *p* < .01 (two-tailed) level

### Belief in Food Addiction: Associated Individual Characteristics

Food addiction symptomology, assessed by the mYFAS, was positively correlated with belief in food addiction (r = .22, *p* < .01) (Table 4). Thus, participants who reported experiencing behavioral indicators of addictive-like eating were more likely to endorse belief in the food addiction construct. Age, gender, political party identification, BMI, and race were not significantly associated with belief in food addiction (all *p*s > .15).

**Table 4:** Correlations Between Belief in Food Addiction (FA) and Continuous Individual Characteristics.

	Belief in FA	Age	BMI	mYFAS Symptom Count
Belief in FA	1	.03	.07	.22**
Age		1	.11	.11
BMI			1	.33**
mYFAS Symptom Count				1

*Correlation is significant at the *p* < .05 (two-tailed) level

**Correlation is significant at the *p* < .01 (two-tailed) level

### Belief in Food Addiction is Associated With Obesity-Related Policy Support

Belief in food addiction was positively related to increased policy support for obesity-related initiatives (F(1,191) = 37.49, *p* < .001, η_p_^2^ = .16). A second model was utilized to examine whether belief in food addiction significantly predicted obesity-related policy support when accounting for BMI, mYFAS symptom count, age, gender, political party identification, and race. Univariate GLM revealed that belief in food addiction was significantly associated with policy support (F(1,175) = 14.37, *p* < .001, η_p_^2^ = .08) even when accounting for other factors potentially associated with obesity-related policy support (BMI, mYFAS symptom count, age, gender, political party identification, and race). In this model, age (F(1,175) = 7.39, *p* < .05, η_p_^2^ = .04) and gender (F(1, 175) = 5.61, *p* < .05, η_p_^2^ = .03) were also significantly related to policy support, with younger individuals and women exhibiting greater support. Further, political party identification was associated with support for obesity-related policy (F(3, 175) = 14.41, *p* < .001, η_p_^2^ = .20), with post-hoc analyses revealing significantly greater policy support for Democrats, relative to Republicans/Libertarians (*p* < .001). BMI, mYFAS symptom count, and race were not significantly associated with policy support in the overall model (all *p*s > .42). Effect sizes for significant variables remained identical when insignificant variables were removed from the model.

**Fig 1:**
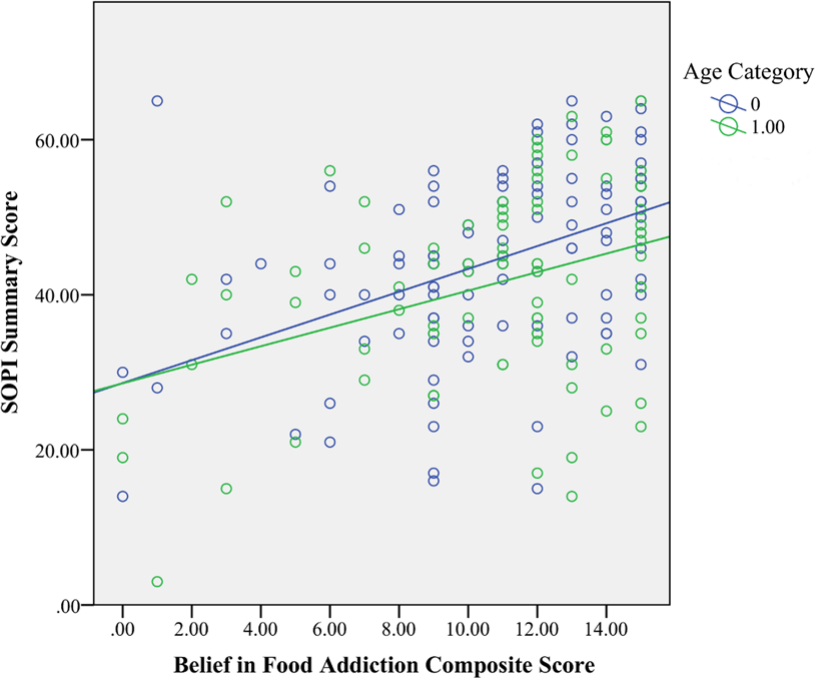
Interaction of Age and Belief in Food Addiction on Obesity-Related Policy Support. (0) Age < 36.95. (1) Age > 39.95.

There was a significant interaction between belief in food addiction with age (F(1,175) = 4.73, *p* < .05, η_p_^2^ = .03). To further explore this moderation, we utilized a median split on age (at 36.95 years old) based on recommendations from Aiken and West [38] regarding how to dissect an interaction when the moderator is a continuous variable. A significant association between belief in food addiction and policy support was observed for both younger (F(1,71) = 22.92, *p* < .001, η_p_^2^ = .19) and older (F(1,71) = 11.55, *p* < .001, η_p_^2^ = .14) individuals, though the effect was stronger for younger participants (Fig 1). For younger individuals, belief in food addiction had nearly double the effect size for predicting obesity-related policy support than political party (belief in food addiction: η_p_^2^ = .19, political party: η_p_^2^ = .10). In contrast, political party had double the effect size than belief in food addiction for older participants (political party: η_p_^2^ = .30, belief in food addiction: η_p_^2^ = .14). Interactions between belief in food addiction with political party and gender were examined but were not significant (all *p*s > 0.11).

## Discussion

Food addiction is a topic of growing public and scientific interest, however, little research has focused on policy implications that may follow from applying an addiction framework to eating-related problems. Given the increase in support for tobacco policies after nicotine was identified as addictive, it is important to consider how belief in food addiction may impact obesity-related policy support. Participants who agreed that certain foods can be addictive were more likely to endorse support for policies that aim to reduce obesity (e.g., fruit/vegetable subsidies, limiting the size of sugar-sweetened beverages). Further, belief in food addiction had a comparable association with policy support as an individual’s policy party identification, which strongly informs support for policy initiatives [39]. Thus, belief in food addiction appears to be an important factor associated with support for the implementation of obesity-related policies and may warrant attention for understanding individual differences in policy support.

Notably, a previous study by Lee and colleagues [16] found no significant association between belief in food addiction and the perceived effectiveness of various policy initiatives. This may suggest that while the current study found belief in food addiction to be a relevant factor in determining support for or belief in the implementation of obesity-related policies, individuals may not differ based on their belief in food addiction for how effective these policies are perceived to be. For example, one may believe that restricting marketing would be an effective treatment for obesity but may not support its implementation if they believe companies have the right to advertise their products. Similarly, education-based interventions (e.g., including calorie labels on menus) were highly supported in the current study (Table 2), but the effectiveness of these approaches to treat obesity is mixed [40–42].

Other individual characteristics also impacted support for obesity-related policies. Consistent with previous studies [31–33], Democrats endorsed significantly higher levels of support than Republicans/Libertarians, suggesting that Democrats are more likely to support government-based initiatives. Also in line with existing research [31–33], women reported greater obesity-related policy support, though this effect was only significant in the model including all individual characteristics. Finally, younger individuals had higher levels of policy support. This finding contrasts another study that reported increased support in older individuals [32], which may be because the current study looks at a broader range of policies (13 relative to 3 policies). Thus, age may be differentially related to policy support based on the types of policies examined, though further research is needed to understand the influence of age on support for obesity-related policy initiatives.

There was also an interaction between age and belief in food addiction in the association with policy support. Belief in food addiction was significantly associated with obesity-related policy support for both younger and older participants, though this effect was greater for younger participants. Among younger individuals, belief in food addiction had almost double the effect size of political party in predicting policy support. In contrast, the opposite was observed for older adults, with political party having twice the effect size than belief in food addiction. Although the concept of food addiction has existed since at least the late twentieth century [43], the idea has recently been receiving greater scientific and public attention. For example, the number of scientific publications on food addiction has increased substantially since 2006 [44]. As a result, the current findings possibly reflect a cohort effect, as younger individuals may have greater exposure to the recent empirical support for food addiction over the course of their life and may be less decided on or engaged with their political party affiliation. On the other hand, older participants may be more established in their political party identification and are being exposed to evidence for the food addiction construct later in life. However, this study is cross-sectional, and longitudinal research is needed to understand whether belief in food addiction and policy support may change throughout development.

The present study had several limitations. Although the current sample was diverse in individual characteristics (e.g., age, BMI, political party identification), Amazon MTurk samples are not nationally representative [34]. The current study had more female participants and fewer African American and Hispanic individuals relative to population data in the United States [45–47]. Thus, it will be important to examine the association of belief in food addiction with policy support in a more representative sample, as demographic variables like gender and race may influence beliefs [32]. Additionally, a measure of political ideology, rather than a single question about self-reported political party identification may be a better indicator of political values in future studies. Further, future studies may consider including both positively and negatively worded questions in order to examine whether an acquiescence bias contributed to the current results [48]. Similarly, psychometric analyses for the measures of belief in food addiction and SOPI were limited to reliability and should be examined further along with indicators of validity in follow-up studies. The association between political party identification and SOPI, with Democrats exhibiting the highest policy support, is consistent with previous literature [31–33] and may provide initial evidence for the convergent validity of the measure. As mentioned previously, the study was cross-sectional in nature, which does not allow for causal attributions to be made. While the current study included individual factors that possibly influence policy support, a third variable may be accounting for both belief in food addiction and obesity-related policy support, such as income level, which appears to be associated with both conceptualizations of food addiction and policy support [17, 31]. Longitudinal studies could more appropriately examine whether changes in the belief in food addiction predicts changes in policy support. Finally, the policies examined were not exhaustive. One important initiative that was not included is the taxation of highly processed foods (e.g., sugar-sweetened beverages), which has been recently implemented in locations like Mexico [49, 50]. Thus, future studies could consider evaluating whether belief in food addiction is related to support for taxation of obesogenic foods or if the association between belief in food addiction and obesity-related policy support varies by policy type (e.g., educational, restrictive).

In summary, belief in food addiction was significantly associated with greater support for obesity-related policies, even when the influences of other relevant factors (e.g., political party) were accounted for. Though applying an addiction, or “brain disease” framework to drugs and behaviors has yielded mixed consequences on stigma [22–24], the current findings suggest that belief in an addiction model of obesity is associated with support for public policies that aim to reduce obesity. Akin to the increased policy initiatives that followed when nicotine was identified as addictive in the 1980s, it is plausible that support for obesity-related policy initiatives would also increase if certain foods were labeled as addictive. However, the timetable of these changes for obesity-related policy is uncertain, as the addictive capacity of nicotine was known for many years prior to the shift in attitudes towards policies to reduce smoking and population level harm (e.g., negative health consequences of second-hand smoke). Finally, further research is necessary to evaluate the food addiction construct, as scientific consensus for the idea has not been reached. Nevertheless, the current study suggests that growing empirical evidence for the contribution of an addictive process to obesity may have important implications for obesity-related policy support.
